# Supplementary material for: Determinants of Pre-Surgical Treatment in Primary Rectal Cancer: A Population-Based Study
Source: Cancers (Basel). 2023 Feb 10;15(4):1154. doi: 10.3390/cancers15041154 (PMC9954598; doi:10.3390/cancers15041154)
Supplement: Supplementary file 1 [file cancers-15-01154-s001.zip › cancers-2186197-supplementary.pdf]

Supplementary tables and figures

Risk groups	2008 Guidelines	2016 Guidelines	2020 Guidelines
**"Good"**	T1-3aN0	T1-2N0<br>T3abN0, Mid/High[1] rectum<br>T2-3bN1, High rectum<br>T3cN0-1, High rectum	T1-3abN0-1, Mid/High rectum, Low rectum (above the intersphincteric space)<br>T3cdN0-1, High rectum<br>T4a[2]<br>T4b easy resection, High rectum<br>N2, High rectum<br>EMVI+, High rectum (above the peritoneal reflection)
**"Bad"**	T3bc<br>N+<br>EMVI+<br>Tumours in the intersphincteric space	T3abN0, Low rectum<br>T2-3bN1, Low/Mid rectum<br>T3cN0-1, Low/Mid rectum<br>T3d<br>N2<br>EMVI+	T1-3abN0-1, Low rectum (in the intersphincteric space)<br>T3cdN0-1, Low/Mid rectum<br>T4a<br>T4b easy resection, Low/Mid rectum<br>N2, Low/Mid rectum<br>EMVI+, Low/Mid rectum, High rectum (not completely above the peritoneal reflection)<br>MRF+ (TD, LN+)
**"Ugly"**	T3d-T4<br>MRF+	T4<br>MRF+	T4b difficult resection<br>MRF+ (primary)[3]<br>Lateral lymph nodes

Figure S1. Group criteria and characteristics according to the three different national guidelines in use during the time. Lymph node positivity, N+; Extramural vessel invasion, EMVI; Mesorectal fascia, MRF; Tumour deposit, TD; Lateral lymph node, LN.

T-stage with subdivision of cT3 into a-d according to the depth of infiltration (<1mm, 1-5 mm, 5-15 mm and >15 mm) and cT4 according to involvement of peritoneum only (a) or other organs (b), in cT3 distance to MRF or the intersphincteric fascia, respectively, described as threatened if <1 mm or positive if involved (collectively designated MRF+), presence of EMVI, engagement of mesorectal lymph nodes (cN1-2) or LN+ (arbitrarily >10 mm). No distinction of whether it was the primary tumour or lymph nodes threatening MRF was made prior to the 2020 guidelines. LN+ was neither considered until in the 2020 guidelines although used as a criterion for inclusion in the RAPIDO/LARCTUS protocols (see Supplementary Table 3). The presence of TD has not been implemented in the guidelines. The possibility to register LN or TD in the quality register SCRCR was not present before 2017. The major differences between the 2008 and the 2016 (used from 2015) guidelines were that fewer patients were included in the bad group (cT3abN0 above 5 cm and cT3abN1 above 10 cm were considered as good) and that lymph node positivity was not solely based upon whether a node was visible or not, but that they should have at least one of the following criteria (size above 5 mm, irregular border or irregular texture), similar to the recent ESGAR guidelines (Beets-Tan et al., 2018). Further, the major differences between the 2016 and 2020 (used from 2019) guidelines were that even fewer stages belonged to the intermediate group. In an evaluation (Hammarström et al, 2019), it was estimated that between 38 and 77% of the rectal cancers were recommended pre-treatment according to 17 different guidelines. According to the Swedish guidelines, principally according to the 2008 version, 73% should be pre-treated. The corresponding figure using the 2020 version is 49%. The formal implementation of national guidelines in Sweden requires a prolonged consultation time and approval of all regions (today 21). Since key persons in the preparatory work of the guidelines were from Uppsala, they came into use a year before they were published.

[1]Tumour height from anal verge measured with a rigid rectoscope: Low 0-4 cm, Mid 5-9 cm, and High rectum 10-15 cm.

[2]T4a with limited spread can be directly operated (= "early/good").

[3]If MRF+ is caused by growth in or against an easily resectable organ/structure, short-course radiotherapy 5x5 Gy can be given (= "intermediate/bad").

Risk groups	Treatment
**“Good”**	Resection surgery/TEM
**“Bad”**	Preoperative RT 5x5 Gy + Immediate/Delayed surgery
**“Ugly”**	Chemoradiotherapy (CRT)[1]

Figure S2. Recommended treatments according to risk groups

[1]In the 2020 national guidelines an alternative to CRT is also suggested according to the RAPIDO/LARCTUS-protocols; 5x5 Gy followed by 3-5 months of chemotherapy before surgery. In elderly, frail persons, scRT with delayed surgery was recommended instead of CRT and chemotherapy was then not recommended in the interval to surgery.

Trial name	Description	Treatment alternatives
**Stockholm III** [1]	A multicentre, randomised, non-blinded, phase 3, non-inferiority trial. 840 patients with non-metastasized non-polypoid and resectable rectal cancer* were recruited and randomised in 1998-2013.	- scRT + immediate surgery<br>- scRT + delayed surgery<br>- Long-course RT + delayed surgery
**RAPIDO** [2]	A multicentre, open label, randomized, controlled, phase 3 trial. 920 patients with locally advanced rectal cancer with high risk for recurrence** were enrolled and randomized in 2011-2016.	- CRT (28 daily fractions of 1·8 Gy up to 50·4 Gy or 25 fractions of 2·0 Gy up to 50·0 Gy + capecitabine)***<br>- scRT (5x5 Gy) + 6 cycles CAPOX or 9 cycles FOLFOX4
**LARCT-US** [3]	A phase 2 trial based on the experience from the RAPIDO trial. Patients with a newly diagnosed rectal cancer at high risk of failing locally and/or systemically have been included in 2016-2020.	- scRT (5x5 Gy) + 4 cycles CAPOX

Figure S3. Description of the two randomised trials and the national phase III trial that patients could participate in. Short-course radiotherapy, scRT; radiotherapy, RT; chemoradiotherapy, CRT; gray, Gy.

**Although many early/good tumours could formally be included in the Stockholm III trials, unofficially during the latter part of the trial, inclusion was restricted to the intermediate/bad group of tumours. Since the Stockholm III trial was still open until January 2013, this meant that some early/good tumours were included and thus pre-treated with RT even if the guidelines from 2008 did not recommend this. Of the M0 patients, 34 were included in Stockholm III, 109 in RAPIDO and 89 in LARCT-US.*

***Primary rectal tumours classified as high risk on pelvic MRI with at least one of the following criteria: clinical tumour (cT) stage cT4a or cT4b, extramural vascular invasion (EMVI+), clinical nodal stage cN2, incolved mesorectal fascia (MRF+), or enlarged lateral lymph nodes.*

****Optional adjuvant CT (8 cycles CAPOX or 12 cycles FOLFOX6) at some sites.*

[1] Erlandsson, J., et al., Optimal fractionation of preoperative radiotherapy and timing to surgery for rectal cancer (Stockholm III): a multicentre, randomised, non-blinded, phase 3, non-inferiority trial. Lancet Oncol, 2017. 18(3): p. 336-346.

[2] Bahadoer RR, et al. Short-course radiotherapy followed by chemotherapy before total mesorectal excision (TME) versus preoperative chemoradiotherapy, TME, and optional adjuvant chemotherapy in locally advanced rectal cancer (RAPIDO): a randomised, open-label, phase 3 trial. Lancet Oncol. 2021 Jan;22(1):29-42. doi: 10.1016/S1470-2045(20)30555-6. Epub 2020 Dec 7. Erratum in: Lancet Oncol. 2021 Feb;22(2):e42. PMID: 33301740.

[3] https://clinicaltrials.gov/ct2/show/NCT03729687.

Table S1. Characteristics at baseline of all patients, both with and without distant metastasis (M0.1)

Characteristics	Uppsala (C-region) ***n* = 700**	Dalarna (W-region) ***n* = 781**	***P-value***
**Age, median**	70 (32-96)	72 (26-96)	**<0.001**
**Men**	407 (58)	467 (60)	0.519
**cT-stage**			0.315
**T1**	40 (6)	51 (7)	
**T2**	108 (15)	135 (17)	
**T3**	345 (49)	356 (46)	
a	*73 (21)*	*59 (17)*	
b	*123 (36)*	*147 (41)*	
c	*101 (29)*	*99 (28)*	
d	*32 (9)*	*37 (10)*	
**T4**	189 (27)	202 (26)	
a	*39 (21)*	*67 (33)*	
b	*143 (76)*	*132 (65)*	
**cN-stage**			**0.002**
**N0**	206 (29)	286 (37)	
**N1**	263 (38)	255 (33)	
**N2**	212 (30)	197 (25)	
**cM-stage**			0.810
**M0**	542 (77)	608 (78)	
**M1**	158 (23)	172 (22)	
**MRF+ (in T3-tumours)**	144 (42)	111 (31)	**0.019**
**EMVI+**	246 (37)	222 (29)	**0.006**
**Tumour level**			0.165
**Low (0–4 cm)**	156 (22)	199 (26)	
**Mid (5–9 cm)**	256 (37)	282 (36)	
**High (10–15 cm)**	285 (41)	298 (38)	

Clinical tumour stage, cT-stage; clinical nodal stage, cN-stage; Mesorectal fascia, MRF; extramural vessel invasion, EMVI.

Table S2. Proportions of EMVI-positive tumours in relation to cT- and cN-stage in M0-patients. Number of patients within parenthesis

cTN-stage	Uppsala	Dalarna
**cT1**	0% (0)	0% (0)
**cT2**	1% (1)	0% (0)
**cT3**	33% (88)	28% (82)
**cT4**	54% (58)	47% (52)
**cN0**	6% (11)	7% (19)
**cN1**	27% (54)	28% (54)
**cN2**	60% (82)	51% (61)

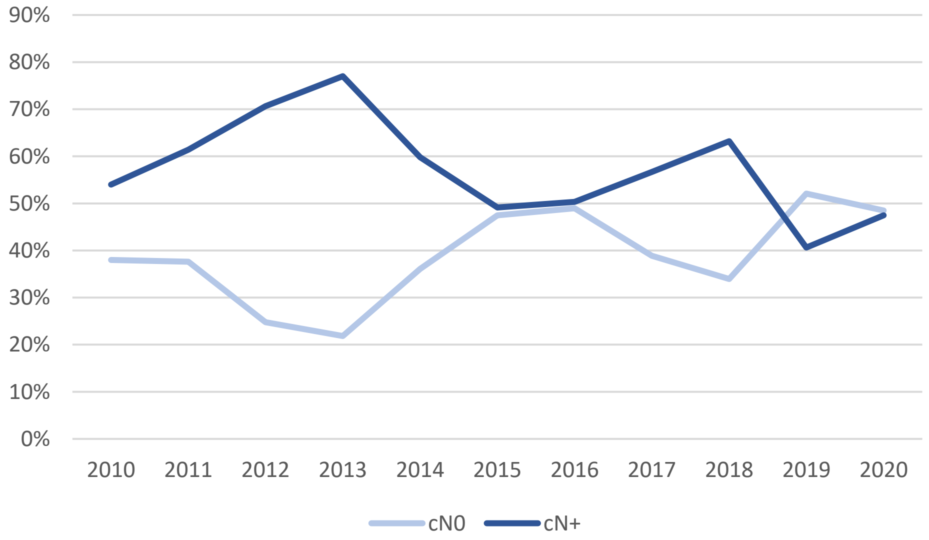

Figure S4. Distribution of cN-stage over time in Uppsala and Dalarna regions. The same patterns were seen in the two regions. The number of patients each year does not add up to 100% since nodal status was not detailed in all patients. No statistical comparisons have been applied to these changes, but the increase from 2010 to 2013 was recognised by members of the MDTs and prompted an initiation of revised criteria for node positivity in the coming guidelines used from before 2015 although the guidelines were not published until 2016. The criteria were stressed even further in the new guidelines used from 2019 (published 2020).

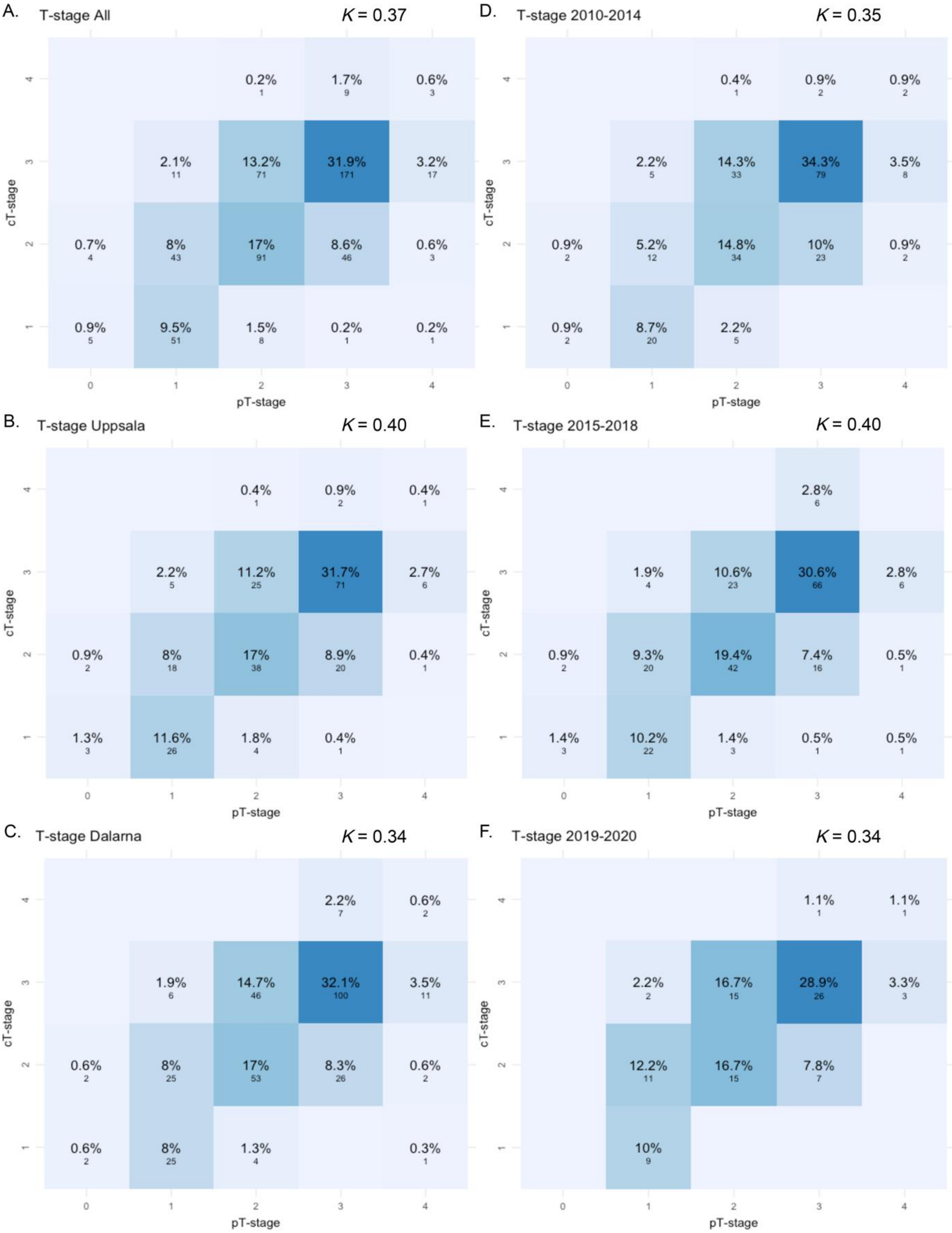

Figure S5. Correlation between clinical and pathological T-stage in patients that had immediate surgery (with or without prior short-course therapy). A) All patients with available c/pT-stage (n=536), B) Uppsala region (n=224), C) Dalarna region (n=312), D) 2010-2014 (n=230), E) 2015-2018 (n=216), F) 2019-2020 (n=90).

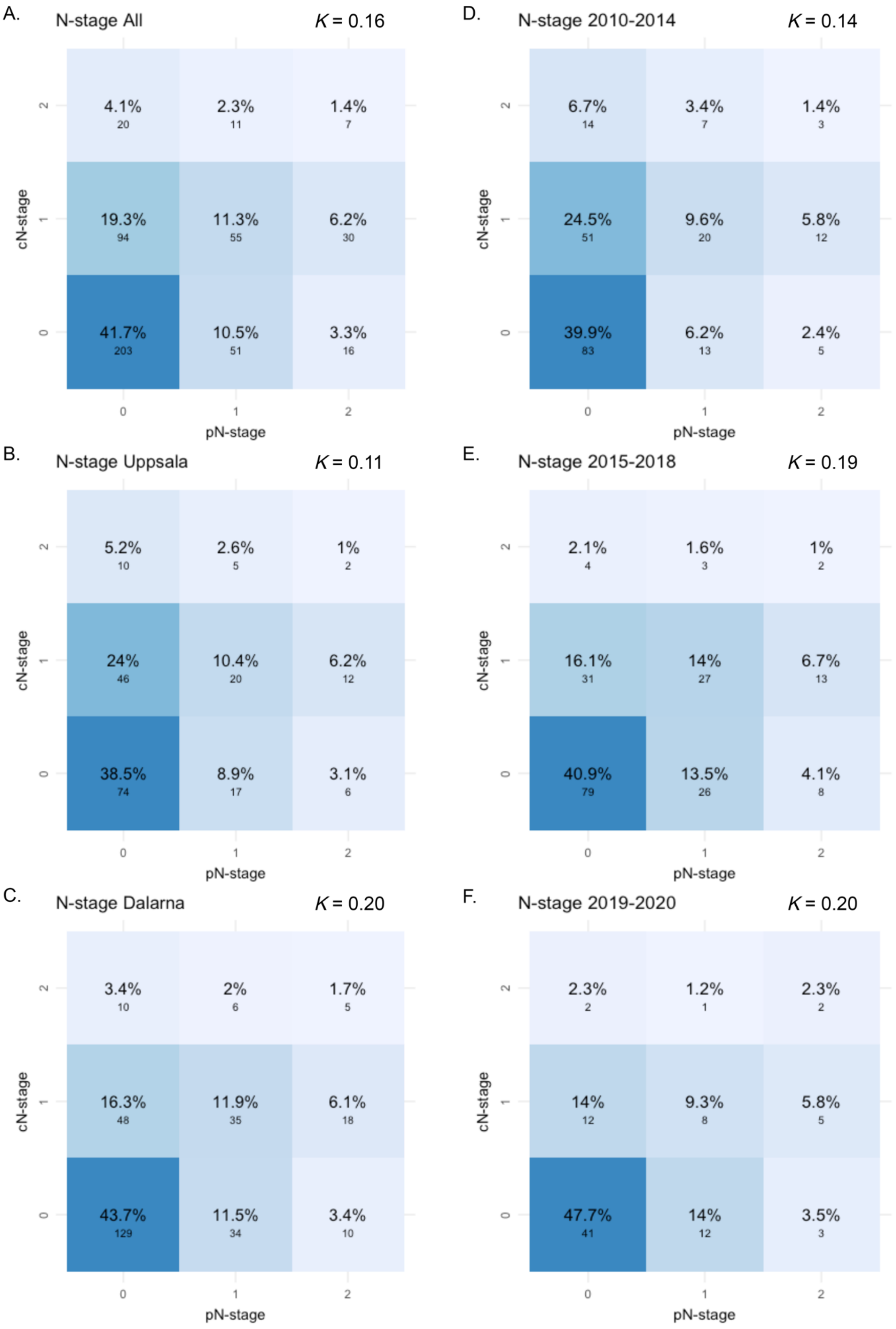

Figure S6. Correlation between clinical and pathological N-stage in patients that had immediate surgery (with or without prior short-course radiotherapy). A) All patients with available c/pN-stage (n=487), B) Uppsala region (n=192), C) Dalarna region (n=295), D) 2010-2014 (n=208), E) 2015-2018 (n=193), F) 2019-2020 (n=86). For all patients, an overestimation of N-stage was done in 31% and an underestimation in 20% of the cases.

Table S3. Characteristics at baseline of patients that had either Immediate surgery (direct surgery or surgery immediately after scRT), left column, where a comparison between cTN and pTN could be made or that had pre-treatment with delayed surgery (scRT+delayed surgery or CRT/scRT+CT), right column, where downstaging/downsizing may influence the comparison

Characteristics	Immediate surgery ***n* = 583**	Pre-treatment ***n* = 522**
**Age, median**	72 (26-93)	70 (29-96)
**Men**	346 (59)	300 (58)
**cT-stage**		
**T1**	80 (14)	5 (1)
**T2**	192 (33)	39 (8)
**T3**	273 (47)	279 (53)
a	*81 (30)*	*39 (14)*
b	*136 (50)*	*98 (35)*
c	*47 (17)*	*107 (38)*
d	*3 (1)*	*29 (10)*
**T4**	14 (2)	195 (37)
a	*11 (79)*	*50 (26)*
b	*3 (21)*	*139 (71)*
**cN-stage**		
**N0**	336 (58)	100 (19)
**N1**	183 (31)	203 (39)
**N2**	38 (7)	215 (41)
**pT-stage***		
**T0**	9 (2)	52 (13)
**T1**	111 (19)	20 (5)
**T2**	175 (30)	89 (21)
**T3**	234 (40)	206 (39)
a	*64 (27)*	*38 (18)*
b	*78 (33)*	*68 (33)*
c	*47 (20)*	*58 (28)*
d	*7 (3)*	*15 (7)*
**T4**	27 (5)	45 (11)
a	*19 (70)*	*16 (36)*
b	*6 (22)*	*22 (49)*
**pN-stage***		
**N0**	326 (56)	286 (69)
**N1**	120 (21)	80 (19)
**N2**	55 (9)	47 (11)
**MRF+ (in cT3-tumours)**	30 (11)	151 (55)
**CRM+****	15 (3)	17 (4)
**Missing**	38 (7)	19 (5)
**EMVI+**	61 (11)	214 (41)
**Tumour level**		
**Low (0–4 cm)**	88 (15)	169 (32)
**Mid (5–9 cm)**	200 (34)	193 (37)
**High (10–15 cm)**	293 (51)	160 (31)
**Risk group**		
**Early/Good**	333 (57)	45 (9)
**Intermediate/Bad**	178 (31)	141 (27)
**Locally advanced/Ugly**	49 (8)	330 (63)

Clinical tumour stage, cT-stage; clinical nodal stage, cN-stage; Mesorectal fascia, MRF; extramural vessel invasion, EMVI.

[*]Among the pre-treated patients, 411 of them underwent surgery (the rest had a watch-and-wait strategy) and had available pathology reports. pN+ means node-positive at baseline, whereas pN0 cannot tell anything about nodal status before treatment. Of 59 operated cN0 tumours, 13 (22%) were pN+, providing a minimum figure for underreporting of node positivity. Of the 125 pN+ tumours, 111 (89%) were cN+ and, thus, accurately staged.

[**]Circumferential resection margin (CRM) is reported in the pathology report. It is considered positive if the distance is 0 or <1 mm.
